# Peripheral blood DNA methylation differences in twin pairs discordant for Alzheimer’s disease

**DOI:** 10.1186/s13148-019-0729-7

**Published:** 2019-09-02

**Authors:** Mikko Konki, Maia Malonzo, Ida K. Karlsson, Noora Lindgren, Bishwa Ghimire, Johannes Smolander, Noora M. Scheinin, Miina Ollikainen, Asta Laiho, Laura L. Elo, Tapio Lönnberg, Matias Röyttä, Nancy L. Pedersen, Jaakko Kaprio, Harri Lähdesmäki, Juha O. Rinne, Riikka J. Lund

**Affiliations:** 1 Turku Bioscience Centre, University of Turku and Åbo Akademi University, FIN-20520 Turku, Finland; 2 Turku Doctoral Programme of Molecular Medicine, University of Turku, FI-20014 Turku, Finland; 3 Department of Computer Science, Aalto University School of Science, FI-00076 Helsinki, Finland; 4 Institute of Gerontology and Aging Research Network-Jönköping (ARN-J), School of Health and Welfare, Jönköping University, SE-55111 Jönköping, Sweden; 5 Department of Medical Epidemiology and Biostatistics, Karolinska Institutet, SE-17177 Stockholm, Sweden; 6 Drug Research Doctoral Program, University of Turku, FI-20014 Turku, Finland; 7 Turku PET Centre, University of Turku, FI-20520 Turku, Finland; 8 Institute for Molecular Medicine Finland, University of Helsinki, FI-00014 Helsinki, Finland; 9 Turku Brain and Mind Center, FinnBrain Birth Cohort Study, Institute of Clinical Medicine, University of Turku, FI-20014 Turku, Finland; 10 Department of Pathology/Neuropathology, Turku University Hospital, University of Turku, FI-20014 Turku, Finland; 11 Department of Psychology, University of Southern California, Los Angeles, CA 90089 USA; 12 Division of Clinical Neurosciences, Turku University Hospital, FI-20014 Turku, Finland; 13 Department of Public Health, University of Helsinki, FI-00271 Helsinki, Finland; 14 Department of Psychiatry, University of Turku and Turku University Hospital, FI-20014 Turku, Finland

**Keywords:** Alzheimer’s disease, DNA methylome, Hippocampus, Peripheral blood, Twin pair

## Abstract

**Background:**

Alzheimer’s disease results from a neurodegenerative process that starts well before the diagnosis can be made. New prognostic or diagnostic markers enabling early intervention into the disease process would be highly valuable. Environmental and lifestyle factors largely modulate the disease risk and may influence the pathogenesis through epigenetic mechanisms, such as DNA methylation. As environmental and lifestyle factors may affect multiple tissues of the body, we hypothesized that the disease-associated DNA methylation signatures are detectable in the peripheral blood of discordant twin pairs.

**Results:**

Comparison of 23 disease discordant Finnish twin pairs with reduced representation bisulfite sequencing revealed peripheral blood DNA methylation differences in 11 genomic regions with at least 15.0% median methylation difference and FDR adjusted *p* value ≤ 0.05. Several of the affected genes are primarily associated with neuronal functions and pathologies and do not display disease-associated differences in gene expression in blood. The DNA methylation mark in *ADARB2* gene was found to be differentially methylated also in the anterior hippocampus, including entorhinal cortex, of non-twin cases and controls. Targeted bisulfite pyrosequencing of the DNA methylation mark in *ADARB2* gene in 62 Finnish and Swedish twin pairs revealed that, in addition to the disease status, DNA methylation of this region is influenced by gender, age, zygosity, *APOE* genotype, and smoking. Further analysis of 120 Swedish twin pairs indicated that this specific DNA methylation mark is not predictive for Alzheimer’s disease and becomes differentially methylated after disease onset.

**Conclusions:**

DNA methylation differences can be detected in the peripheral blood of twin pairs discordant for Alzheimer’s disease. These DNA methylation signatures may have value as disease markers and provide insights into the molecular mechanisms of pathogenesis. We found no evidence that the DNA methylation marks would be associated with gene expression in blood. Further studies are needed to elucidate the potential importance of the associated genes in neuronal functions and to validate the prognostic or diagnostic value of the individual marks or marker panels.

**Electronic supplementary material:**

The online version of this article (10.1186/s13148-019-0729-7) contains supplementary material, which is available to authorized users.

## Introduction

Alzheimer’s disease (AD) is an aging-associated neurodegenerative disorder and the most common cause of dementia. The molecular mechanisms of AD are not known in detail. The disease is characterized by accumulation of beta-amyloid plaques and hyperphosphorylated tau protein in the brain tissue. The pathological changes can start decades before the first clinical symptoms appear [1–3]. In the early-onset form of AD, which accounts for approximately 2–10% of the cases, the symptoms can start already before age of 30 years. In 5–10% of the early onset cases, the disease is caused by autosomal dominant mutations in the genes *APP*, *PSEN1*, and/or *PSEN2* [1, 4]. The majority of AD cases are of the late-onset form, which typically manifests after 65 years of age [1, 4]. Based on twin studies, the estimated heritability of the disease exceeds 50% [5]. Genetic association with apolipoprotein E (*APOE*) epsilon 4 (*ε4*) allele is common in both early- and late-onset form. Furthermore, genome-wide association studies have identified over 20 additional variants each contributing less than 1% to the heritability of liability. Together with *APOE ε4*, these known variants explain approximately 29% of the heritability [4, 6–8] leaving a large fraction unexplained.

In addition to genetic factors, the morbidity of AD is influenced by modifiable lifestyle factors, such as physical activity and cognitive ability, nutrition, alcohol use, and smoking [1, 9]. These factors may influence the disease process through epigenetic mechanisms, such as DNA methylation, by causing changes detectable in various tissues. Such changes may provide insights into the molecular mechanisms of the disease and have potential as informative biomarkers. DNA methylation marks associated with AD have been discovered in several brain regions [10–14]. Whether similar marks exist in peripheral blood is still unclear [15]. The question is complicated by the fact that methylation levels at CpG sites associated with the disease may be driven by genetic variants. Within-pair comparison of disease-discordant monozygotic (MZ) twin pairs with, in practice, identical genomes, the same age and sex, and who share many intrauterine and early-life environmental factors provides a powerful approach for detection of the disease-associated methylation sites [16]. Dizygotic (DZ) disease-discordant twin pairs also provide improved sensitivity to the analyses as they share on average 50% of their genome and are of the same age and matched for prenatal and shared rearing environmental factors.

Modern technologies, such as positron emission tomography (PET), enable monitoring of the disease status in the brains of living subjects and are already utilized in diagnostics together with clinical examination and biomarkers to identify the individuals with first symptoms [9, 17, 18]. However, novel high-throughput methods enabling early detection and large scale monitoring of the disease status would be highly valuable. The aim of this study was to identify epigenetic marks associated with late-onset AD in peripheral blood by comparing DNA methylation profiles of the disease-discordant twin pairs and to examine overlap in brain tissue. To evaluate potential as prognostic or diagnostic marker, one of the most interesting loci was selected for validation in extended twin cohorts from Finland and Sweden.

## Results

### Twin pairs discordant for Alzheimer’s disease have similar genetic risk load for the disease

Before identification of the epigenetic marks, the genetic risk load of the twin pairs for Alzheimer’s disease was characterized. To measure the genetic risk load for AD in the Finnish study subjects, we analyzed the *APOE* genotypes and 21 loci previously associated with AD as a risk or protective variants (Table 2, Additional file 1) [4, 6]. In this analysis, we included 9 full MZ and 12 full DZ AD-discordant twin pairs, 9 unrelated controls, and 18 unrelated cases (Table 1). Monozygotic co-twins were confirmed to have identical single nucleotide polymorphism (SNP) profiles. All dizygotic co-twins were identical for the *DSG2*, *CASS4*, and *SORL1* alleles but carried differences in 2–10 other variants. Most of the twin pairs (17 of 23 pairs) had *APOE* ε3ε3 genotype. Standardized GRS were calculated for the study groups based on the 21 loci previously associated with AD (Table 2). According to the generalized linear regression analysis, the GRS was not associated with the disease status (Wald test *z* value < 1, Pr(>[z]) > 0.5).

**Table 1:** Characteristics of the samples utilized in the study

	Controls			Cases	
Study and sample type	No of subjects (male/female)	Age range (years)		No of subjects (male/female)	Age range (years)
Finnish cohort (*n* = 97)					
Peripheral blood (*n* = 93)					
ADD^a^ MZ twin pairs	12/9	67–86		12/9	67–86
ADD DZ twin pairs	6/6	70–72		6/6	70–72
Unrelated individuals	1/8	61–77		9/9	70–86
Peripheral blood mononuclear cells (*n* = 4)					
MZ twin pairs discordant for episodic memory	2/0	76		2/0	76
Swedish cohort (*n* = 298)					
Peripheral blood (*n* = 298)					
ADD MZ twin pairs	5/5	62–83		5/5	63-83
ADD DZ twin pairs	11/8	63–84		11/8	63–84
Incident^b^ ADD MZ twin pairs	15/23	62–85		15/23	62–85
Incident ADD DZ twin pairs	33/49	53–88		33/49	53–88
NIH NeuroBioBank (*n* = 12)					
Anterior hippocampus with entorhinal cortex (*n* = 12)					
Unrelated individuals	4/2	56–89		4/2	57–90
Total (*n* = 407)	89/110	53–90		97/111	53–90

^a^Alzheimer’s disease-discordant

^b^Sample collected 0.5–18.5 years before disease onset

**Table 2:** Genetic risk load for Alzheimer’s disease in the Finnish twin pairs and unrelated individuals

				Minor allele frequencies						
SNP	Minor/major allele	Closest gene	Log-OR (*p* ≤ 1.0×10^−4^)^a^	Meta-analysis^a^	MZ twin pairs (*n* = 9)	DZ twin controls (*n* = 12)	DZ twin cases (*n* = 12)	Unrelated controls (*n* = 9)	Unrelated cases (*n* = 18)	All (*n* = 62)
rs10498633	G/T	SLC24A4-RIN3	− 0.094	0.22	0.22	0.21	0.17	0.17	0.17	0.18
rs10792832	G/A	PICALM	− 0.140	0.36	0.44	0.5	0.58	0.39	0.42	0.47
rs10838725	T/C	CELF1	0.079	0.32	0.28	0.29	0.29	0.33	0.31	0.3
rs10948363	A/G	CD2AP	0.095	0.27	0.22	0.08	0	0.22	0.11	0.12
rs11218343	T/C	SORL1	− 0.262	0.04	0.00	0	0	0	0	0
rs11771145	G/A	EPHA1	− 0.102	0.34	0.28	0.42	0.42	0.33	0.22	0.32
rs1476679	T/C	ZCWPW1	− 0.089	0.29	0.44	0.33	0.37	0.33	0.39	0.37
rs17125944	T/C	FERMT2	0.132	0.09	0.11	0.08	0.17	0.22	0.11	0.13
rs190982	A/G	MEF2C	− 0.076	0.41	0.44	0.33	0.29	0.33	0.28	0.32
rs2718058	A/G	NME8	− 0.077	0.37	0.33	0.17	0.29	0.44	0.19	0.27
rs28834970	T/C	PTK2B	0.100	0.37	0.44	0.29	0.21	0.39	0.31	0.32
rs35349669	C/T	INPP5D	0.076	0.49	0.50	0.58	0.62	0.11	0.53	0.49
rs3865444	C/A	CD33	− 0.067	0.31	0.11	0.42	0.33	0.33	0.36	0.32
rs4147929	G/A	ABCA7	0.143	0.19	0.06	0.17	0.12	0.11	0.14	0.12
rs6656401	G/A	CR1	0.167	0.20	0.22	0.42	0.33	0.33	0.22	0.3
rs6733839	C/T	BIN1	0.197	0.41	0.33	0.54	0.37	0.56	0.36	0.42
rs7274581	T/C	CASS4	− 0.132	0.08	0	0	0	0	0	0
rs8093731	C/T	DSG2	− 0.316	0.02	0	0	0	0	0	0
rs9271192	A/C	HLA-DRB5/-DRB1	0.108	0.28	0.33	0.29	0.42	0.33	0.56	0.41
rs9331896	T/C	CLU	− 0.146	0.38	0.22	0.58	0.42	0.28	0.5	0.42
rs983392 GRS^b^	A/G	MS4A6A	− 0.108	0.40	0.39 − 0.16	0.42 − 0.27	0.33 − 0.39	0.61 − 0.28	0.39 − 0.03	0.42 − 0.36

^a^From Lambert et al.

^b^Median standardized genetic risk score

### Blood DNA methylation marks associated with Alzheimer’s disease in the Finnish twin cohort

**Fig. 1:**
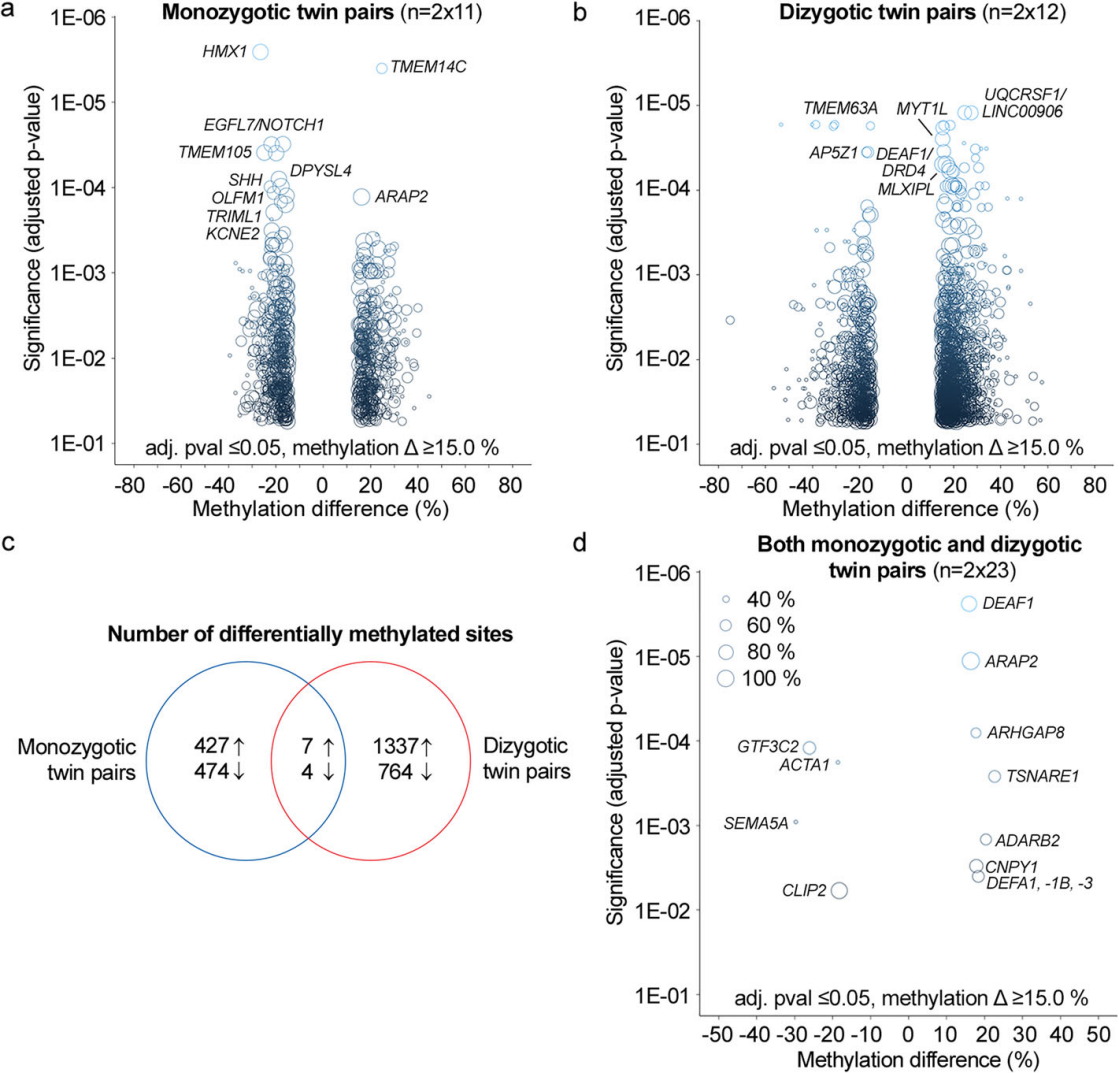
Peripheral blood CpG methylation differences in Finnish twin pairs discordant for Alzheimer’s disease. Peripheral blood CpG methylomes of 11 monozygotic (MZ) and 12 dizygotic (DZ) Finnish twin pairs discordant for Alzheimer’s disease (AD) were profiled with reduced representation bisulfite sequencing. Differentially methylated sites associated with AD in autosomes were identified using RADMeth algorithm and ± 15.0% average methylation difference and B-H FDR adjusted *p* value cut off 0.05 in **a** MZ and **b** DZ twin pairs separately. **c** Differentially methylated sites detected in both MZ and DZ twin pairs were extracted to identify overlaps between the AD-associated sites detected in both groups (Additional file 2). **d** Combined median methylation difference and adjusted *p* value (adj. pval) distribution of these 11 regions associated with AD in both MZ and DZ twin pairs is illustrated. The size of the dot indicates the percentage of the twin pairs, which had the required ≥ 10× coverage in the region. The closest or overlapping gene is indicated for the selected differentially methylated regions

Our first research question was whether DNA methylation marks associated with AD can be detected in the peripheral blood of disease discordant twin pairs. The null hypothesis was that peripheral blood DNA methylation is not associated with the disease status. To address this hypothesis, we first identified the within-pair methylation differences associated with AD in 11 MZ twin pairs with reduced representation bisulfite sequencing (RRBS) as described in the “Methods” section (Table 1). This comparison revealed 427 CpG sites with increased and 474 sites with decreased methylation in AD cases when using cut off values of at least 15.0% median methylation difference and Benjamin-Hochberg false discovery rate (B-H FDR) adjusted *p* value ≤ 0.05 (Fig. 1a, Additional file 2a). Similarly, CpG methylome differences were then identified in 12 DZ twin pairs discordant for AD. This comparison revealed 1337 sites with increased and 764 sites with decreased methylation in AD cases (Fig. 1b, Additional file 2b). We then examined the overlap of differentially methylated CpG sites detected in both MZ and DZ within-pair comparisons. This revealed 11 common sites with consistent methylation difference present in both zygosity groups (adjusted *p* value ≤ 0.05), (Fig. 1c, d, Additional file 2c and Additional file 3). The genes nearest or overlapping with these regions included *DEFA1* (intron), *TSNARE1* (intron), *DEAF1* (intron), *ARAP2* (− 2,197,329 bp from TSS), *CNPY1* (+ 764 bp from TSS), *ADARB2* (exon), *ARHGAP8* (intron), *GTF3C2* (− 1157 bp from TSS), *ACTA1* (+ 29543 bp from TSS), *SEMA5A* (+ 694751 bp from TSS), and *CLIP2* (+ 121538 bp from TSS). No methylation differences were detected in the mitochondrial DNA or X chromosome with the chosen filtering criteria. In summary, DNA methylation changes in 11 genomic regions were found to be associated with AD in peripheral blood of AD-discordant Finnish twin pairs. These regions are henceforth referred to as AD-associated loci in blood. Based on these results, we could reject the first null hypothesis for the 11 CpG sites.

### Overlap of DNA methylation marks associated with Alzheimer’s disease in the anterior hippocampus and peripheral blood

**Fig. 2:**
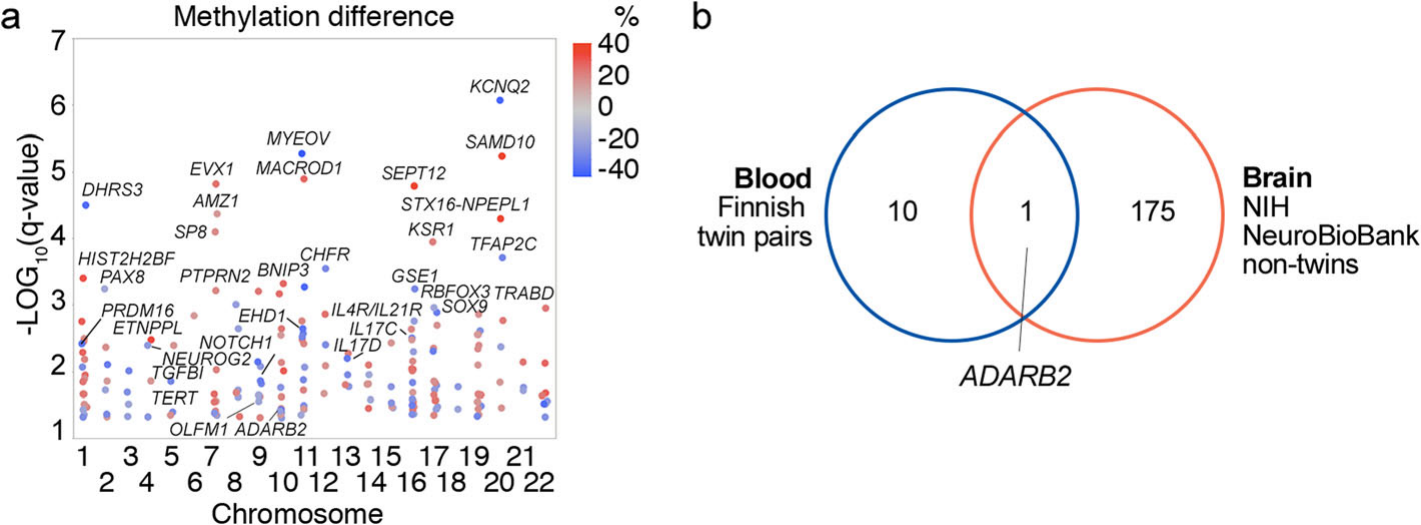
Differentially methylated sites associated with Alzheimer’s disease in the anterior hippocampus. DNA methylation profiles of anterior hippocampus samples of 10 non-twin subjects, including six references and four with Alzheimer’s disease (AD), were examined with reduced representation bisulfite sequencing. The samples were from NIH NeuroBioBank. **a** Differentially methylated sites (201 CpGs) were identified with MethylKit package in R by using a minimum of 15% methylation difference and *q* value below 0.05 as filtering cutoff (Additional file 2d). The 201 differentially methylated CpG sites overlapped or were closest to 176 genes (AD-associated genes in the brain). Examples of the genes are shown. **b** Overlap of genes with AD-associated DNA methylation marks in both peripheral blood and anterior hippocampus of the brain

Our second research question was whether the DNA methylation marks associated with AD in peripheral blood are also detectable in brain tissue. Our null hypothesis was that DNA methylation marks associated with AD in the anterior hippocampus, including the entorhinal cortex, do not overlap with those detected in peripheral blood. Anterior hippocampus, including entorhinal cortex, was selected for analysis, as these brain regions are crucial for memory formation and learning. Both regions were analyzed together as they were not available separately from the biobank. To test the hypothesis, the DNA methylation was profiled with the RRBS from postmortem tissue sections collected from six cases with AD and six controls (Table 1). Two cases were excluded from the final analysis as one was identified to be an outlier in the principal component analysis, and according to the neuropathological examination, one sample was from amygdala. Comparison of cases and controls with MethylKit R package revealed increased methylation at 114 sites and decreased methylation at 87 genomic sites among AD cases (at least 15.0% methylation difference, SLIM adjusted *q* value ≤ 0.05), overlapping or closest to the 176 gene identifiers, in the anterior hippocampus (Fig. 2a). These sites are henceforth referred to as AD-associated loci in the brain. Comparison of the gene identifiers closest to the AD-associated loci in the brain to those detected in blood revealed one overlapping gene *ADARB2* (Fig. 2b). In the gene *ADARB2*, the differentially methylated sites in blood and hippocampus were localized within the same region chr10:1404752-1405717 in exon 3, in the proximity of the exon 3-intron junction. Therefore, the second null hypothesis was rejected.

We also examined the correlation of AD-associated loci in blood with the brain tissue in the publicly available Illumina 450K data by using the online tool https://epigenetics.essex.ac.uk/bloodbrain/. Only two of the AD-associated loci in blood identified in this study are detected by Illumina 450K arrays, including probe cg26376030 in *DEAF1* gene and cg22909083 in *DEFA1B* gene locus. The peripheral blood DNA methylation of probe cg22909083 had high correlation (> 0.95, *p* < 0.05) with all the four regions of brain available in the database, including prefrontal cortex, entorhinal cortex, superior temporal gyrus, and cerebellum. The probe cg26376030 had modest correlation (> 0.26, *p* < 0.05) with prefrontal cortex, entorhinal cortex, and superior temporal gyrus (Additional file 4).

### Gene expression levels in whole blood and individual lymphocytes

Our third research question was whether the genes closest to DNA methylation marks in the blood are also associated with differences in gene expression in peripheral blood from AD cases compared to controls. Our null hypothesis was that the expression of the genes closest to the AD-associated loci in blood is not associated with AD. To address this hypothesis, expression of the 11 genes overlapping or closest to the AD-associated loci (Fig. 1d, Additional file 2c) were examined in the data from whole blood (GSE63061, Europeans) of 122 controls and 121 cases with AD [19] available at the GEO database. Expression of 9 genes was detected; however, there were no differences between AD cases and controls (Additional file 5). Expression of these genes was further examined in individual peripheral blood mononuclear cells (PBMCs) of two twin pairs discordant for episodic memory, one of which were also discordant for mild AD. The proportions of blood cell types expressing these genes were similar between the co-twins and no major differences were detected in the expression of the genes within the twin pairs (Additional files 6 and 7). In conclusion, analysis of blood did not provide evidence that the genes would display major disease associated functional significance in the PBMC populations. Based on our data, we could not reject the third null hypothesis.

### Targeted validation of disease-associated methylation changes in *ADARB2* gene in Finnish and Swedish twin pairs discordant for AD

**Fig. 3:**
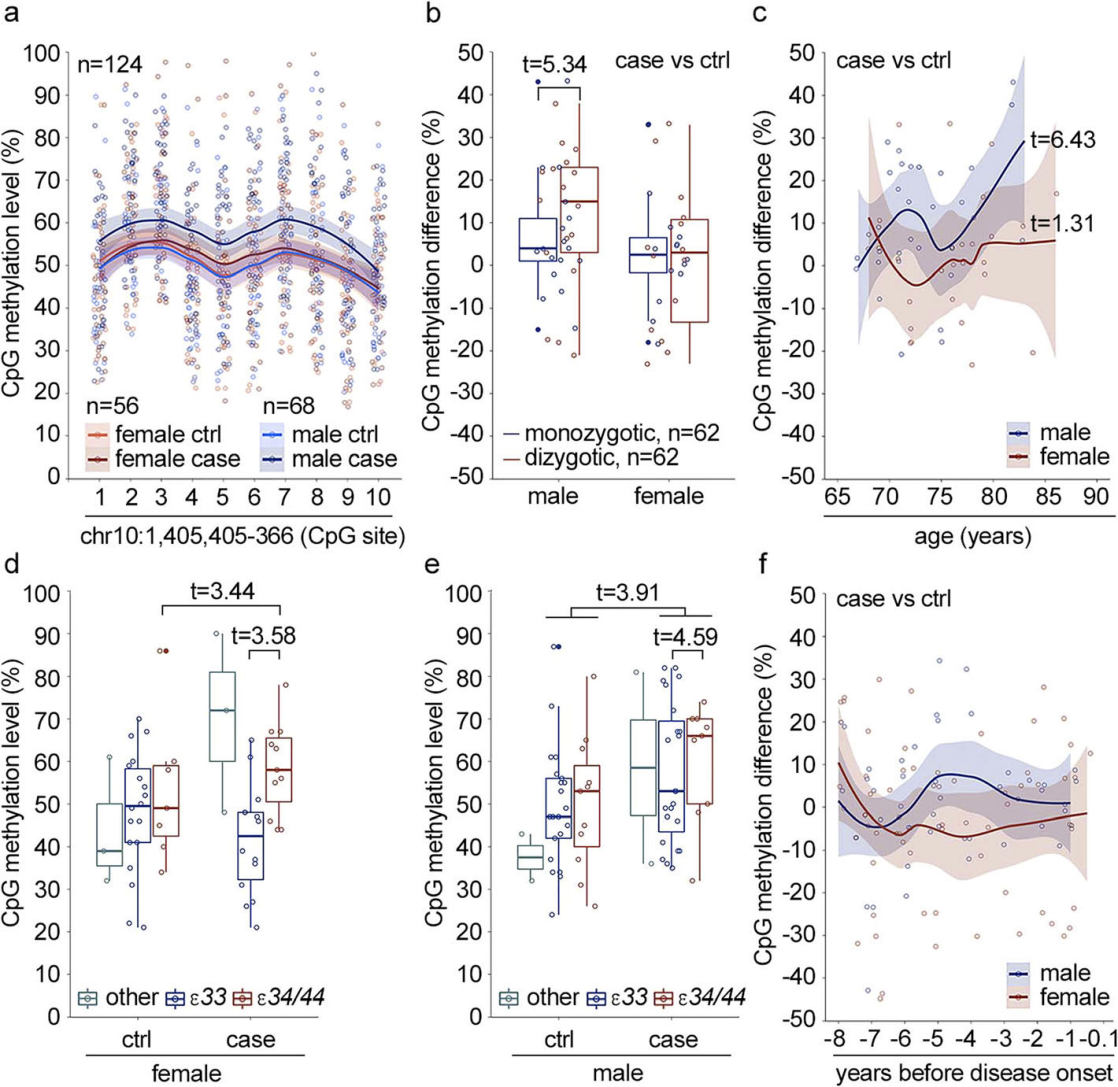
The DNA methylation status of the *ADARB2* gene is associated with Alzheimer’s disease. The DNA methylation level of region chr10:1,405,405-366 in exon 3 of *ADARB2* gene was measured with targeted pyrosequencing in blood DNA samples collected from Finnish (33 pairs) and Swedish (29 pairs) twin pairs discordant for Alzheimer’s disease (AD). The association of the disease status with DNA methylation was examined with linear mixed effects model including gender, zygosity, age, and interaction terms as fixed effects and twin pair information nested with genomic position as a random effect. The country of origin was not associated with the CpG methylation level and was excluded from the final models. **a** The CpG methylation level in male and female controls (ctrl) and cases. Wald *t* value for disease association including both genders is 4.68, for males 6.10, and for females 1.17. **b** Influence of zygosity of CpG methylation difference between twin pairs discordant for AD. **c** Influence of age on the CpG methylation difference between AD discordant twin pairs. Influence of *APOE* genotype on the CpG methylation level was examined separately including only gender as a covariate. Only group ε33 and combined groups ε34/44 were included in the statistical analysis. The CpG methylation levels for **d** female and **e** male ctrls and cases in different *APOE* groups. **f** CpG methylation difference in AD discordant twin pairs before onset of the diseases (not predictive for disease outcome based on Cox mixed effects model). In **b**–**f**, the representative data for CpG site 6 in the region is shown, and in **a**, **c**, and **f**, the lines are the smoothed conditional means with 0.95 confidence intervals area

Our fourth research question was whether the DNA methylation mark in the *ADARB2* gene can be validated with targeted bisulfite pyrosequencing in an extended cohort including an independent twin cohort from Sweden. The null hypothesis was that the DNA methylation mark in *ADARB2* is not associated with AD in an extended cohort. To address this hypothesis, the CpG methylation of the region (chr10:1,405,405-366) in *ADARB2* gene was analyzed with targeted bisulfite pyrosequencing in 62 of AD discordant twin pairs including 33 Finnish and 29 Swedish twin pairs. Association of the CpG methylation with AD was analyzed using linear mixed effects model (lme) [20]. Initially, several models were compared with ANOVA including or excluding zygosity, age, and gender as fixed effects and twin pair identification number nested with genomic position and country of origin as random effects. The model with lowest AIC value was selected as the final model: methylation level (%) ~ disease status + zygosity + age + gender + diseases status × zygosity + disease status × age + disease status × gender + (1 | twin pair identification number/genomic position). Influence of *APOE* genotype (*ε34/ε44* and *ε33)* was examined separately, including disease status and gender as fixed effects, to ensure sufficient number of observations in each subgroup. The influence of *APOE* genotype was examined because it is a well-known risk factor for AD and can influence the progression and severity of the disease, which in turn may be reflected in the state of the DNA methylation marks associated with the disease. Increased CpG methylation level of the region chr10:1,405,405-366 was validated to be associated with AD (Wald *t* value 4.68) and was influenced by interaction with gender, zygosity, and age. Separate examination by sex revealed that the CpG methylation level was increased in male cases (Wald *t* value 6.10) (Fig. 3a, Additional file 8). The methylation difference of the region was higher in DZ than MZ twin pairs (estimate 7.67%, standard error (SE) 1.44, Wald *t* value 5.34) (Fig. 3b), indicating that the association is influenced by genetic factors. In addition, in males the CpG methylation difference between discordant twin pairs increased with age (estimate 1.00% per year, SE 0.15, *t* value 6.43) (Fig. 3c). Interestingly, in females, the CpG methylation was increased in cases with APOE *ε*34/*ε*44 genotype (Wald *t* value 3.44); however, it was not increased in cases with the *ε*33 genotype (Wald *t* value − 1.67) (Fig. 3d). In male cases, the CpG methylation level was higher among cases than controls in both *APOE* genotype groups (Wald *t* value > 3.83), and in male cases, the level in *APOE* ε34/ε44 group was higher in comparison to the male cases with *ε*33 genotype (Wald *t* value 4.59). Of note, visual examination revealed that also the male cases with *ε*33 genotype had polarized into high and low/intermediate CpG methylation level groups (Fig. 3e).

Next, we determined whether the association of the methylation mark with AD is attenuated and if also smoking history is included as a fix effect and interaction term in the linear mixed effects model. Smoking data was available for 60 twin pairs, including 49 current or past smokers and 15 smoking discordant pairs. The association of the methylation mark with the disease status was not attenuated when smoking was included in the analysis (estimate 64.51%, SE 9.23, and Wald *t* value 6.99). However, the smoking did interact with the disease (estimate 8.10%, SE 1.43, and Wald *t* value 5.68). The median methylation level of the mark was higher in those cases who were current or past smokers in comparison to controls and cases who had never smoked or controls who were current or past smokers (Additional file 9). The association with the disease remained significant also in the model with adjustment for *APOE* genotype (estimate 35.41%, SE 9.41, and Wald *t* = 3.76).

In conclusion, our findings from targeted analysis confirm the association of CpG methylation in region chr10:1,405,405-366 with AD and the methylation is influenced by gender, age, zygosity, *APOE* genotype, and smoking. Thus, based on our results, the fourth null hypothesis was rejected.

### CpG methylation status of region chr10:1,405,405-366 in *ADARB2* gene does not predict the disease outcome

Our fifth and last research question was whether the DNA methylation mark in *ADARB2* is predictive for AD. The null hypothesis was that the DNA methylation mark in *ADARB2* gene is not associated with incident AD. To address this hypothesis, the methylation level in region chr10:1,405,405-366 was analyzed with targeted bisulfite sequencing and Cox mixed effects model was used to examine the association of CpG methylation with disease outcome in samples collected 0.5–18.5 years before disease onset from 120 discordant twin pairs from Sweden. The gender and age at blood draw were included as fixed effects in the analysis and relatedness nested with genomic position as a random effect. According to the results, the disease outcome was not associated with the DNA methylation level of the region before disease onset (hazard ratio = 1.00, 95% confidence interval = 1.00–1.00, *p* = 0.63). Thus, the fifth null hypothesis was not rejected (Fig. 3f).

## Discussion

Our results show that differences in DNA methylation associated with AD can be detected in the peripheral blood in at least 11 genomic regions. Although the functional importance of the affected regions in transcriptional regulation requires further studies, our results suggest that the DNA methylation marks are not associated with gene expression in peripheral blood. Several of these genes have been previously linked to neurological functions or pathologies. Among the most interesting findings was the AD-associated methylation signature in the exon 3 of *ADARB2* gene, which was detected in both peripheral blood and anterior hippocampus in brain tissue. Based on previous studies, expression of *ADARB2* is brain specific; however, its function has not been fully characterized. *ADARB2* may have a function in the inhibition of RNA editing by ADAR1 and ADAR2 proteins [21–23]. Importantly, a recent study showed that mice lacking the corresponding exon 3 of *ADARB2* gene display impaired hippocampus-dependent memory formation, learning, and regulation of genes implicated in synaptic functions [24]. Consistently, a variant in *ADARB2* gene has been associated with accelerated cognitive decline after conversion from mild cognitive impairment (MCI) to AD [25]. With targeted validation including independent twin pairs discordant for AD from both Finnish and Swedish cohorts, we further found that the methylation level in *ADARB2* was associated with *APOE* genotype, age, gender, and smoking which all also influence risk of AD. Although these results together suggest potential importance of *ADARB2* in the molecular pathology of AD, the high inter-individual variance and relatively small within-pair differences in blood suggest that methylation at this region alone is unlikely to have value as a diagnostic marker. Furthermore, CpG methylation in *ADARB2* was not predictive for AD when measured before disease onset.

Similarly to *ADARB2,* several of the differentially methylated regions in blood overlapped or were closest to genes that are highly expressed in the brain or have been previously linked to neuronal functions or pathologies, such as neurogenesis, synaptic function, cognitive decline, intellectual disability, social interaction, schizophrenia, and Parkinson’s and Alzheimer’s diseases [24, 26–31]. For example, increased levels of alpha defensins (encoded by *DEFA1*) have been reported in the CSF of patients with AD [29], whereas mutations in *DEAF1* gene can cause severe intellectual disability and behavioral problems. Conditional knockout of *Deaf1* gene in mouse model leads to a phenotype with impaired memory and increased anxiety [26]. *TSNARE1* gene has been previously associated with schizophrenia and rate of cognitive decline in late MCI [32–34].

Why DNA methylation marks associated with neurodegenerative disease can be detected in the blood is not fully clear. One potential explanation is that these marks are caused by environmental or lifestyle factors, which affect the methylation status of DNA also in peripheral blood; however, the influence on the disease process manifests only in the specific tissues where the affected genes are functionally active or important. Nonetheless, potential importance of these genes in the neuronal functions and disease process remains to be elucidated. Further studies are also needed to elucidate whether combinations of differentially methylated regions in multiple AD-associated loci would increase sensitivity in distinguishing AD cases from cognitively preserved controls and to evaluate the value of these changes as predictive disease markers.

Previously, two independent large-scale studies on different regions of the brain have found DNA methylation marks in the *ANK1* and *RHBDF2* genes to be associated with AD [11, 12]. Both studies utilized Illumina 450K arrays. These regions were not captured by the RRBS method utilized in our study. Vice versa, the differentially methylated region in *ADARB2* gene found in this study is not covered by the probes present in 450K array.

A limitation in our study is that no adjustment for smoking was performed in the RRBS analysis due to the small number of samples for this variable available in the discovery cohort. To our knowledge, the genes nearest to the differentially methylated sites in peripheral blood have not been previously associated with smoking, suggesting that the results were not confounded by this factor. Another limitation is that the analysis was carried out by using whole blood and cell type heterogeneity can potentially confound the results. The adjustment of the RRBS results with the Bacon method is expected to correct for potential bias caused by cell type heterogeneity.

## Conclusions

Our results show that DNA methylation differences can be detected in the peripheral blood of twin pairs discordant for Alzheimer’s disease. These DNA methylation signatures may have value as disease marker candidates and may provide insights into the molecular mechanisms of pathogenesis. Our results suggest that the DNA methylation marks do not associate with gene expression in blood. Further studies are needed to elucidate the functional importance of the affected genes and to validate the prognostic or diagnostic value of the individual marks or marker panels.

## Methods

### Study participants and samples

A total of 395 peripheral blood samples from Finnish and Swedish cohorts and 12 brain samples from NIH NeuroBioBank were analyzed in this study (Table 1). In more detail, the older Finnish twin cohort [35, 36] of 2483 individuals, born 1922–1937, had been previously screened for cognitive functions by phone interview [37], and the discordant twin pairs were invited to neuropsychological testing, brain magnetic resonance imaging, PET amyloid imaging [18, 38–41], and collection of EDTA blood samples. Based on the overall evaluation, a diagnosis of AD was made (29 pairs). The controls were not required to have negative PET amyloid imaging. In addition to the original cohort, four twin pairs born in 1915–1950 were included in the study. Furthermore, BD Vacutainer® CPT™ samples were collected from two MZ twin pairs (age 76 years) discordant for episodic memory, where one pair was also discordant for mild AD. These two twin pairs were from a younger Finnish twin cohort (born in 1938–1944). The blood DNA samples from Swedish twin pairs (149 pairs), born 1907–1953, were from the Swedish Twin Registry [42] and had been collected before (120 pairs) or after (29 pairs) the onset of the disease. The participants originated from three sub-studies of aging within the Swedish Twin Registry [42], namely the Swedish Adoption/Twin Study of Aging (SATSA) [43], the Study of Dementia in Swedish Twins (HARMONY) [44], and TwinGene [42]. Disease information was available through linkage to nationwide health registers for all Swedish twins, and in addition, AD was clinically evaluated as part of the SATSA and HARMONY study [45]. Furthermore, our study material included fresh frozen post-mortem brain tissue from anterior hippocampus, including dentate gyrus and entorhinal cortex. The samples had been collected from 12 adults (57–90 years), including six patients with AD and six non-demented controls. Five of the individuals were white and seven had unknown ethnic origin.

### Nucleic acid extractions

The DNA was extracted from EDTA blood with Qiagen QIAamp DNA Blood kit and from fresh frozen tissue with QIAamp DNA Micro kit. Qubit 3.0 dsDNA HS Assay, Thermo Scientific NanoDrop 2000, and Fragment Analyzer HS Genomic DNA assay (Advanced Analytical) were used for the quality analysis and quantitation.

### Targeted genotyping and variant data analysis

Genotyping of *APOE* alleles and 21 SNPs previously associated with AD [6] (Table 2) was performed with Illumina TruSeq Custom Amplicon assay and sequencing (500 cycles) with Illumina MiSeq. Illumina BaseSpace was used for variant analysis. Genetic risk scores were calculated based on 21 genetic variants by summing up the number of risk or protective alleles, weighted by the effect size from the stage 1 and 2 meta-analyses in a genome-wide association study of AD [6]. Influence of the GRS on the disease outcome was examined with generalized linear model in R version 3.4.3 [46]. The influence of sex, age, and *APOE* genotype on the model was examined separately due to small number of samples. Packages lmtest [47], multiwayvcov [48], and fmsb [49] were utilized in the model and in calculation of Nagelkerke’s *R* square.

### Reduced representation bisulfite sequencing and data analysis

The libraries for RRBS were prepared as previously described [50, 51] and were sequenced (1 × 50bp) with Illumina HiSeq2500/3000. The trimmed reads (Trim Galore v0.4.1 [52] were mapped to hg19 (blood) or hg38 (brain) reference genome using Bismark v0.14.5/0.15.0 [53]. The differentially methylated sites in blood were identified using a paired comparison with RADMeth [54] using CpG sites with coverage ≥ 10× in both twins in a pair, and at least four pairs per study group, as an input. The genomic inflation factors and bias caused by unobserved factors were calculated and corrected with BACON v1.10.1 package [55]. The genomic inflation factor was 1.07 (bias 0.30) for MZ and 1.14 (bias 0.50) for DZ twins. We expect this step to correct for any bias caused by cell type heterogeneity. The differentially methylated regions were filtered according to Benjamin-Hochberg false discovery rate adjusted *p* value combined with RADMeth algorithm (< 0.05) followed by absolute average within-pair methylation difference (≥ 15.0%) and central interval (70% interval < 0 or 70% interval > 0) for these sites. Due to small cohort size, the analysis was not adjusted for smoking. Information of the smoking history of the twin pairs is provided in the Additional file 9. The effect sizes of the top-ranked differentially methylated sites from the monozygotic twin pairs (“discovery set”) were examined for reproducibility in the dizygotic twin pairs (“validation set”) by comparing the corresponding values in a scatterplot. The effect size was measured as methylation difference (% estimate by RADMeth) and the standardized effect size was measured as estimated methylation difference over standard deviation of pairwise methylation. Also, for brain samples, the CpG sites with coverage ≥ 10× and less than 99.9th percentile of coverage in each sample were included. Methylkit v.1.1.7 [56] was used to detect differentially methylated CpG sites with a minimum and consistent difference of 15% and *q* value ≤ 0.05 as a cutoff. MethylKit uses the SLIM method to adjust *p* values to *q* values. The bisulfite conversion efficiency of all the samples was above 99% according to the lambda spike-in control.

### Targeted bisulfite pyrosequencing analysis

The PCR primers 5′-gtaatttagtggtgttgttgaat-3′, 5′-biotin-cctaacccccaaccaacttcttactac-3′ and the sequencing primer 5′-gggttgagttaagtgtgtttggtaga-3′ for the region chr10:1405336-1405409 (hg19) were designed with Qiagen PyroMark AssayDesign SW 2.0. The samples were prepared with Qiagen EpiTect Fast Bisulfite Conversion and Qiagen PyroMark PCR kits and sequenced with Qiagen PyroMark Q24 Advanced system. The amplicons were analyzed with PyroMark Q24 Advanced software version 3.0.0. The statistical analysis was carried in R v3.4.3 [46] using packages lme4 v1.1-15 [20], car v2.1-6 [57], survival 2.42-4 [58], coxme 2.2-10 [59], and ggplot2 v2.2.1 [60]. Association of AD with CpG methylation was examined with lme4 package, with modeling methylation level as a function of disease status. Influence of covariates and their interaction with AD in the model was examined, including gender, age, zygosity, *APOE* genotype, and smoking (never, ever) as fixed effects and twin pair nested with genomic position and country of origin as random effects as specified in the results. The model with the lowest AIC was chosen. The Cox mixed effects model with coxme package was utilized to examine CpG methylation level as a prognostic marker of disease outcome. In this model, only age and gender were included as fixed effects and twin pair information nested with genomic position as a random effect.

### Single-cell transcriptome analysis

PBMCs were isolated from the BD Vacutainer® CPT™ Cell Preparation tubes. Single-cell RNA sequencing (scRNA-seq) libraries were prepared using Chromium^TM^ controller, Single Cell 3′ Reagents kit (10x Genomics), targeting at the recovery of 3000 cells per sample, and were sequenced with Hiseq2500. The data was preprocessed using Cell Ranger v. 1.2.0 (10x Genomics) and the GRCh38 genome reference. The filtered gene-barcode unique molecular identifier count matrix of the aggregated sample (Cell Ranger aggr tool) was normalized using a global-scaling normalization from the Seurat R package v. 1.4.0.9 [61]. Finally, the full Seurat scRNA-seq analysis was performed for each sample individually. Cells with unique gene count over 1750 or proportion of mitochondrial genes over 10% were first filtered out. Ten most significant principal components were selected for the graph-based clustering, the different PBMC cell types were identified using canonical markers, and the cell type frequencies were estimated. Paired *t* test was used to test differential expression between sample groups.

### Public data used

The data from GSE63061 [19] available at NCBI GEO database was analyzed with GEO2R using default settings [62, 63].

## Additional files

Additional file 1: Table of *APOE* genotypes of the study individuals. (XLSX 10 kb)

Additional file 2: Table of differentially methylated sites associated with Alzheimer’s disease. Differentially methylated sites in Alzheimer’s disease discordant a) monozygotic, b) dizygotic and in c) both twin pair groups and d) in brain of non-twin subjects with Alzheimer’s disease in comparison to reference subjects. (XLSX 415 kb)

Additional file 3: The effect sizes of the top-ranked differentially methylated sites. The effect sizes of the top-ranked differentially methylated sites from the monozygotic twin pairs (MZ, "Discovery set") were examined for reproducibility in the dizygotic twin pairs ("DZ, Validation set") by comparing the corresponding values in a scatterplot. A) effect size was measured as methylation difference (% estimate by RADMeth) and b) standardised effect size was measured as estimated methylation difference over standard deviation of pairwise methylation difference (n = 172, R = 0.452, *p* value = 4.754e-10 and R = 0.354, *p* value = 1.837e-6, respectively, by Pearson correlation test). The top 200 sites in the MZ cohort were initially selected (ranked by *q* value) of which 172 met the coverage criteria for the DZ cohort. The correlation tests indicate overall consistency in direction of methylation of the top-ranked MZ sites in the DZ cohort. (TIF 4938 kb)

Additional file 4: Correlation of the DNA methylation in blood and brain. Correlation of two peripheral blood DNA methylation marks associated Alzheimer’s disease was examined in publicly available Illumina 450K data available from blood and different regions of brain by using the online tool: https://epigenetics.essex.ac.uk/bloodbrain/. (PDF 1782 kb)

Additional file 5: Gene expression level of candidate genes in blood of non-twin subjects with Alzheimer’s disease in comparison to reference subjects. Gene expression levels of the candidate genes from Additional file 2c in GEO NCBI data from blood (GSE63061) of non-twin subjects with Alzheimer’s disease in comparison to reference subjects. (XLSX 12 kb)

Additional file 6: Proportion of peripheral blood mononuclear cell subtypes in twin pairs discordant for episodic memory as determined by single cell 3' RNA-sequencing. (PDF 4 kb)

Additional file 7: Expression levels of the candidate genes in the peripheral blood mononuclear cell subtypes in twin pairs discordant for episodic memory as determined by single cell 3' RNA-sequencing. (TIF 37322 kb)

Additional file 8: Residual plots for linear mixed effects models. CpG methylation of the region chr10:1,405,405-366, in exon three of *ADARB2* gene was analyzed with targeted pyrosequencing. Association of disease status with methylation level (outcome) was examined with linear mixed effects model (lme4 R package) including zygosity, age and gender as fixed effects and twin pair information nested with genomic position as random effects. In the Figure is the representative data of the residual plots for the models: a) me ~ dis + zyg + age + sex + dis * zyg + dis * age + dis * sex + (1 | pairid/pos) in the data including both male and female twin pairs, b) me ~ dis + zyg + age + dis * zyg + dis * age + (1 | pairid/pos) in the data including only males. me = CpG methylation level, dis = disease status, zyg = zygosity, pairid = twin pair information, pos = genomic position. (JPG 297 kb)

Additional file 9: DNA methylation level of the *ADARB2* mark is higher in the Alzheimer’s disease cases with a history of smoking. Smoking history was included in the linear mixed effects model as a fixed effect, and interaction term, to determine whether this adjustment attenuates association of the *ADARB2* DNA methylation mark with Alzheimer’s disease. The analysis was carried out by including all the 60 twin pairs with smoking data available. According to the results the disease association remained significant also when smoking was included in the model (estimate 64.51%, SE 9.23, Wald *t* value 6.99, not adjusted for *APOE* genotype). Smoking did interact with the disease status (estimate 8.10%, SE 1.43, Wald *t* value 5.68) and the median methylation level was increased in the cases with a smoking history in comparison to non-smoking cases and controls as well as controls with a smoking history. The association remained significant also when *APOE* genotype was included in the model (estimate 35.41%, SE 9.41 and Wald *t* value 3.76). A) DNA methylation levels for the *ADARB2* mark in non-smokers and past or current smokers are shown separately for females with *APOE ε33* genotype who differ from the other groups and show no disease association. B) Residual plot for the full model: me ~ dis + dis * zyg + dis * age + dis * sex + dis * smo + dis * apoe + (1 | pairid/pos). me = CpG methylation level, dis = disease status, zyg = zygosity, smo = smoking history (never, ever), apoe = apoe genotype group, pairid = twin pair information, pos = genomic position. (PDF 715 kb)

## Abbreviations

AD: Alzheimer’s disease
ADD: Alzheimer’s disease-discordant
APOE: Apolipoprotein E (APOE)
DZ: Dizygotic
GRS: Genetic risk score
MCI: Mild cognitive impairment
MZ: Monozygotic
PBMC: Peripheral blood mononuclear cells
PET: Positron emission tomography
RRBS: Reduced representation bisulfite sequencing
scRNA-seq: Single-cell RNA sequencing
SE: Standard error
SNP: Single nucleotide polymorphism
