# Supplementary figures and images for: Peripheral blood DNA methylation differences in twin pairs discordant for Alzheimer’s disease
Source: Clin Epigenetics. 2019 Sep 2;11:130. doi: 10.1186/s13148-019-0729-7 (PMC6721173; doi:10.1186/s13148-019-0729-7)

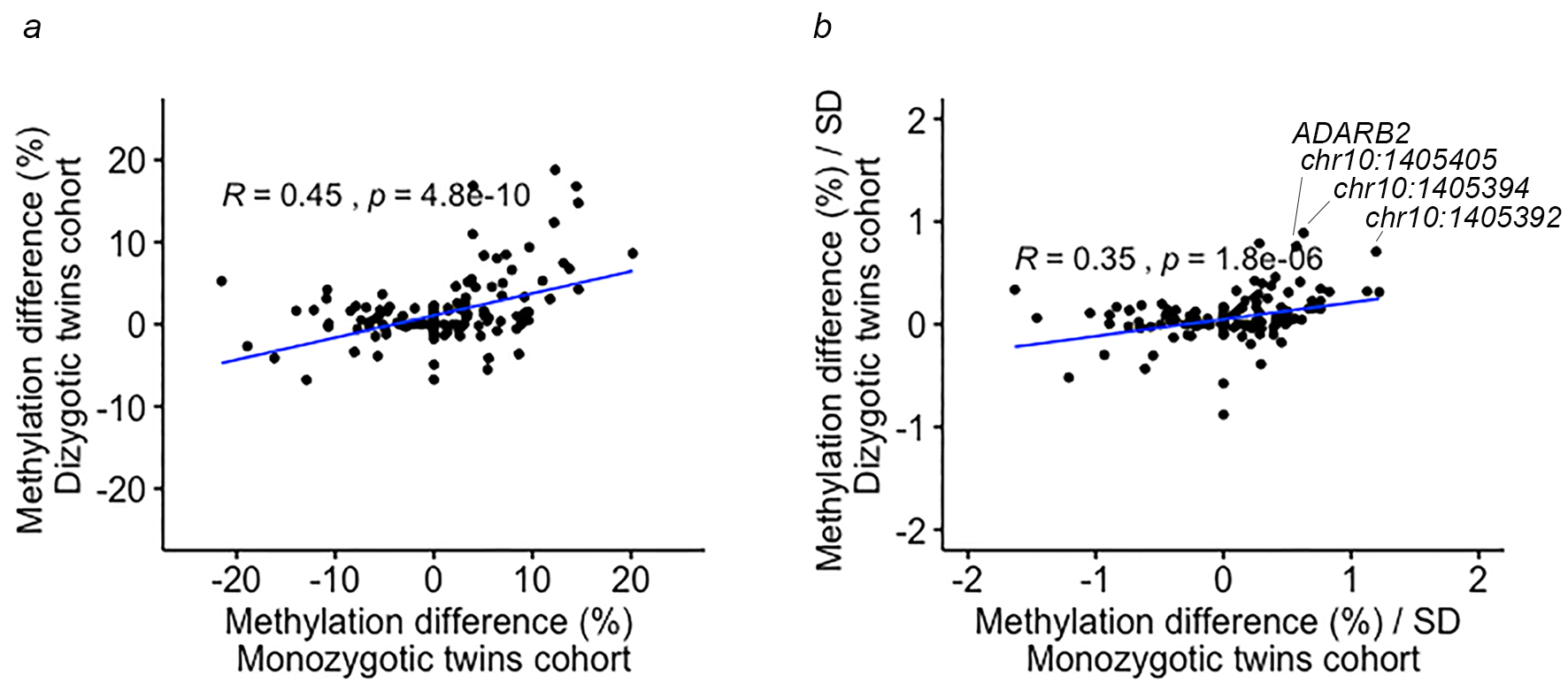

Supplement: Supplementary file 3 — The effect sizes of the top-ranked differentially methylated sites. The effect sizes of the top-ranked differentially methylated sites from the monozygotic twin pairs (MZ, "Discovery set") were examined for reproducibility in the dizygotic twin pairs ("DZ, Validation set") by comparing the corresponding values in a scatterplot. A) effect size was measured as methylation difference (% estimate by RADMeth) and b) standardised effect size was measured as estimated methylation difference over standard deviation of pairwise methylation difference (n = 172, R = 0.452, p value = 4.754e-10 and R = 0.354, p value = 1.837e-6, respectively, by Pearson correlation test). The top 200 sites in the MZ cohort were initially selected (ranked by q value) of which 172 met the coverage criteria for the DZ cohort. The correlation tests indicate overall consistency in direction of methylation of the top-ranked MZ sites in the DZ cohort. (TIF 4938 kb) [file 13148_2019_729_MOESM3_ESM.tif]

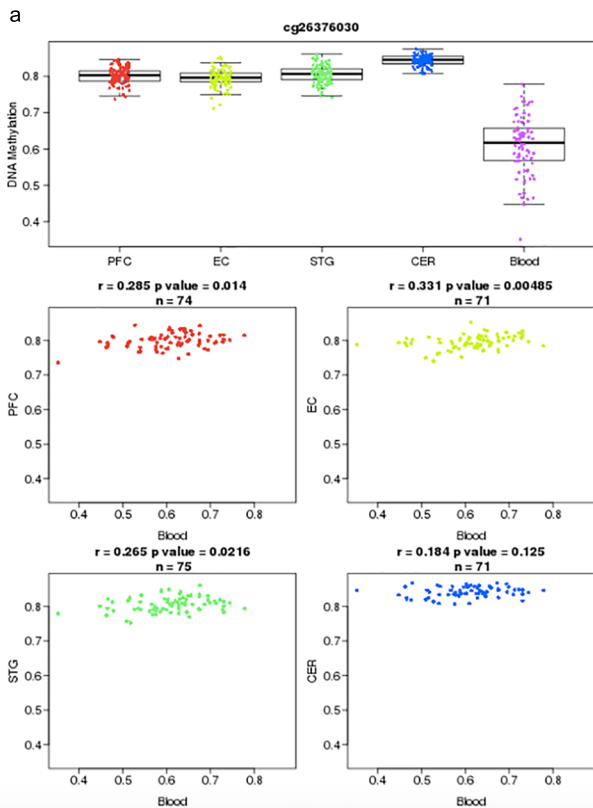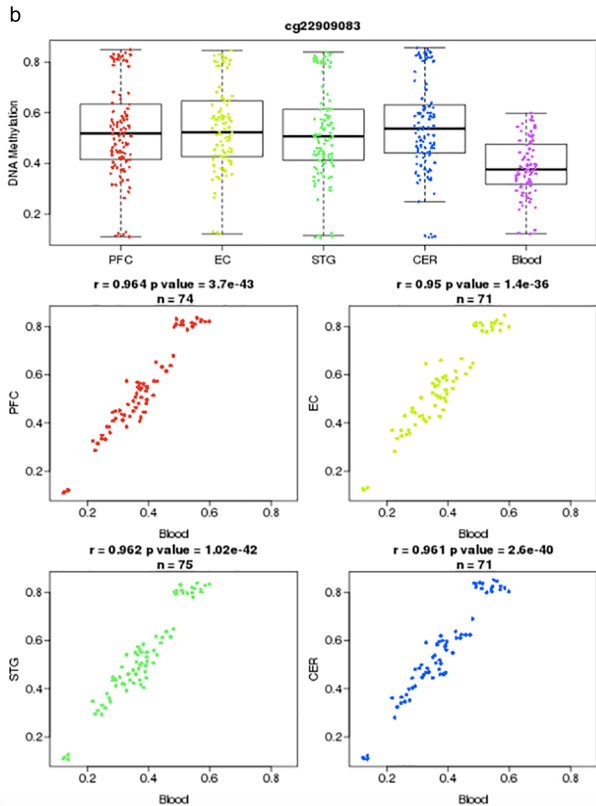

Supplement: Supplementary file 4 — Correlation of the DNA methylation in blood and brain. Correlation of two peripheral blood DNA methylation marks associated Alzheimer’s disease was examined in publicly available Illumina 450K data available from blood and different regions of brain by using the online tool: https://epigenetics.essex.ac.uk/bloodbrain/. (PDF 1782 kb) [file 13148_2019_729_MOESM4_ESM.pdf]

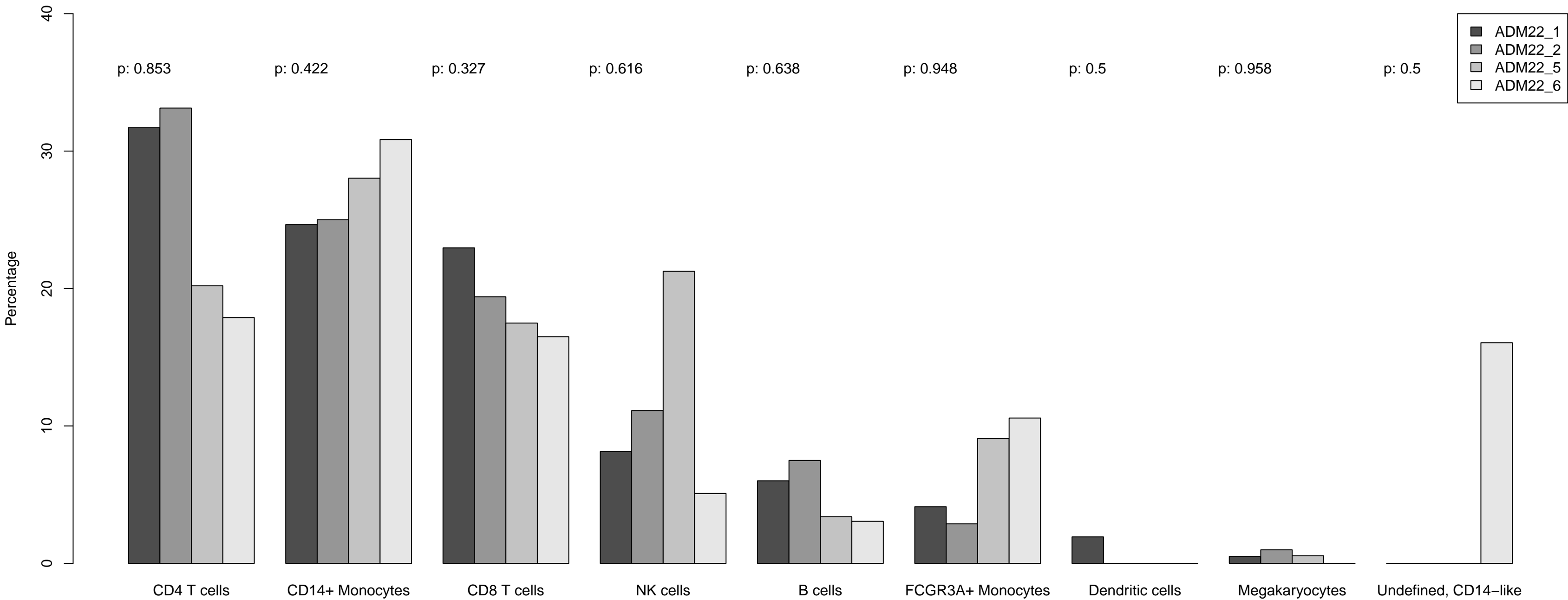

Supplement: Supplementary file 6 — Proportion of peripheral blood mononuclear cell subtypes in twin pairs discordant for episodic memory as determined by single cell 3' RNA-sequencing. (PDF 4 kb) [file 13148_2019_729_MOESM6_ESM.pdf]

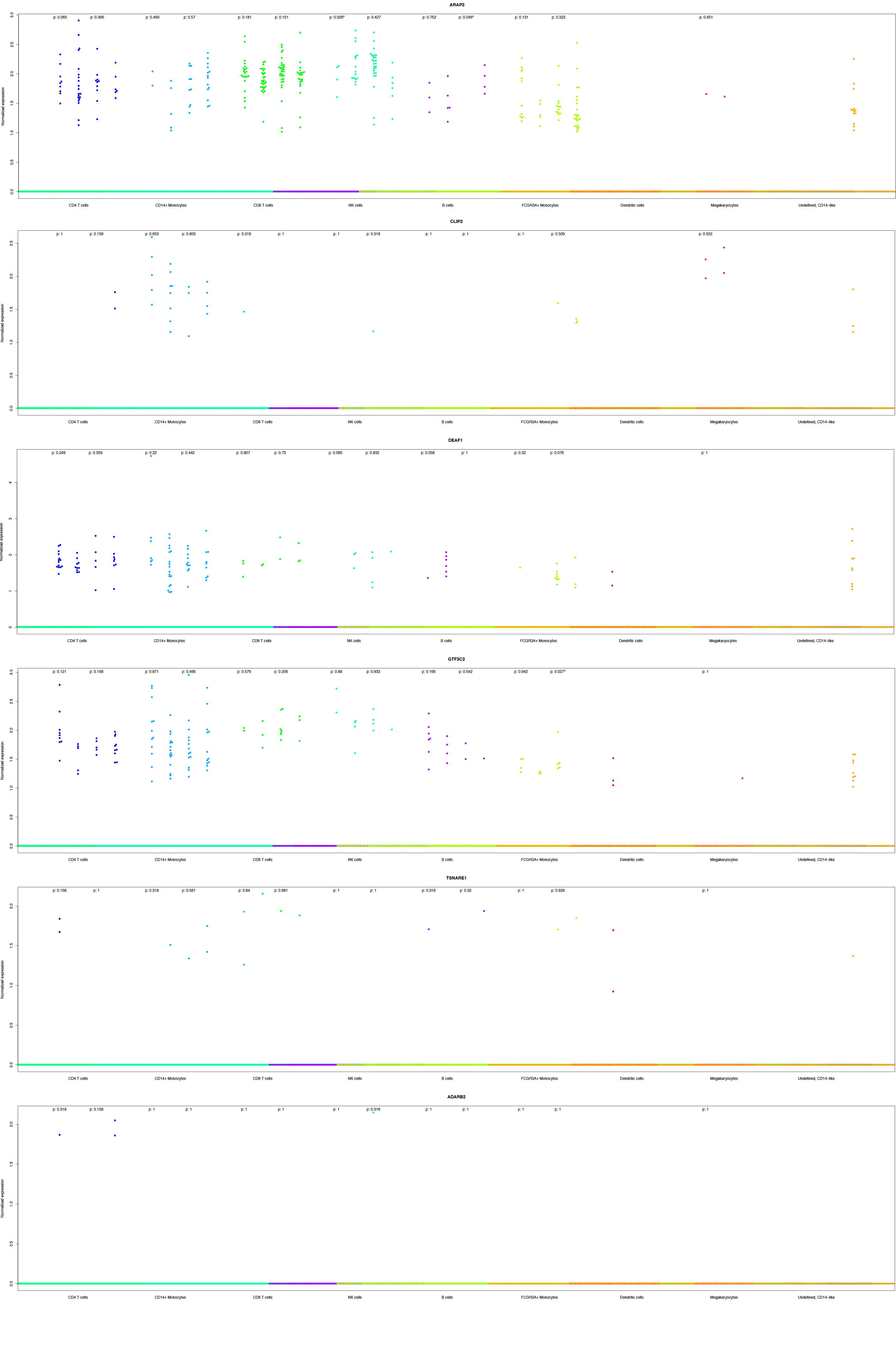

Supplement: Supplementary file 7 — Expression levels of the candidate genes in the peripheral blood mononuclear cell subtypes in twin pairs discordant for episodic memory as determined by single cell 3' RNA-sequencing. (TIF 37322 kb) [file 13148_2019_729_MOESM7_ESM.tif]

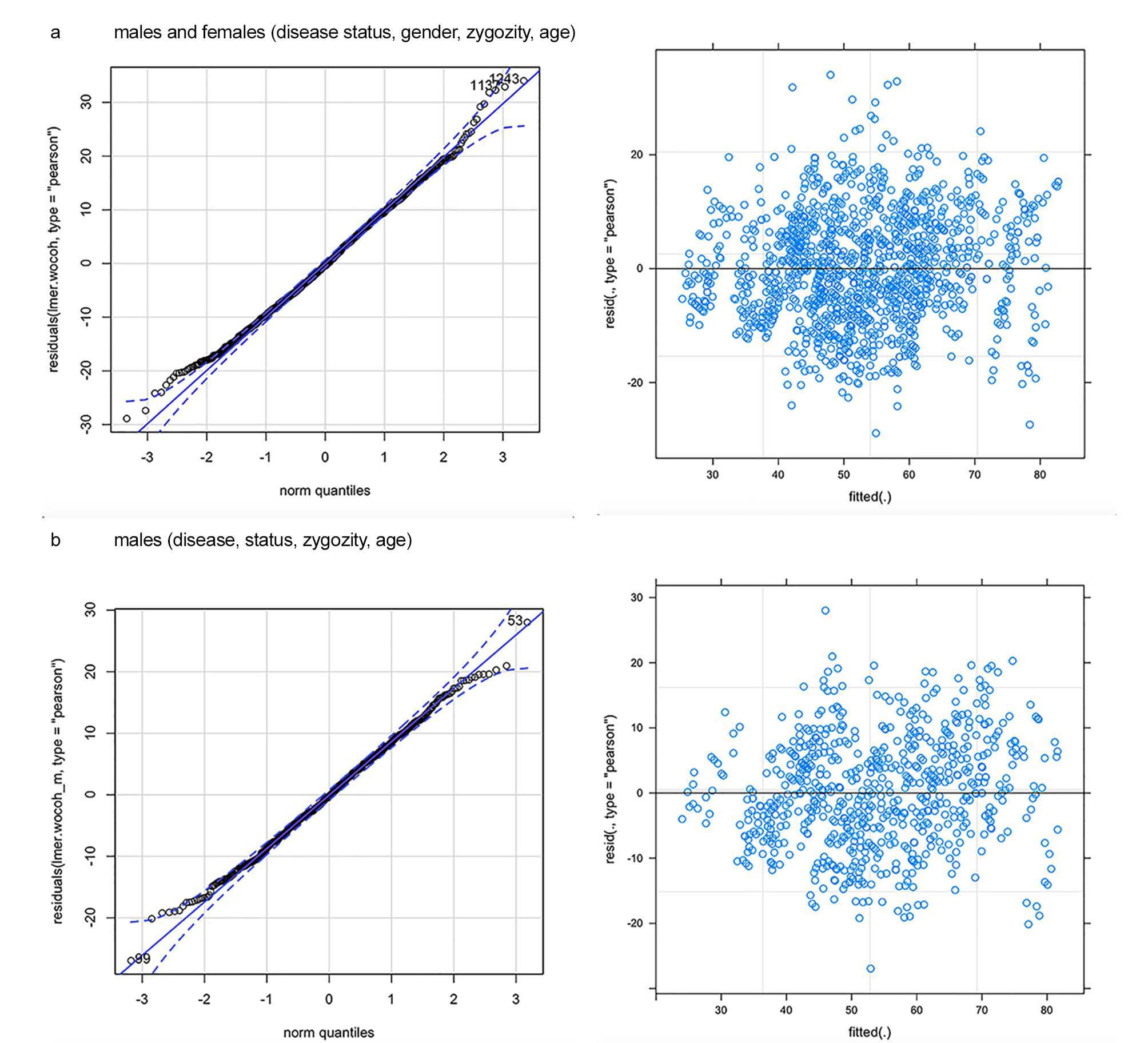

Supplement: Supplementary file 8 — Residual plots for linear mixed effects models. CpG methylation of the region chr10:1,405,405-366, in exon three of ADARB2 gene was analyzed with targeted pyrosequencing. Association of disease status with methylation level (outcome) was examined with linear mixed effects model (lme4 R package) including zygosity, age and gender as fixed effects and twin pair information nested with genomic position as random effects. In the Figure is the representative data of the residual plots for the models: a) me ~ dis + zyg + age + sex + dis * zyg + dis * age + dis * sex + (1 | pairid/pos) in the data including both male and female twin pairs, b) me ~ dis + zyg + age + dis * zyg + dis * age + (1 | pairid/pos) in the data including only males. me = CpG methylation level, dis = disease status, zyg = zygosity, pairid = twin pair information, pos = genomic position. (JPG 297 kb) [file 13148_2019_729_MOESM8_ESM.jpg]

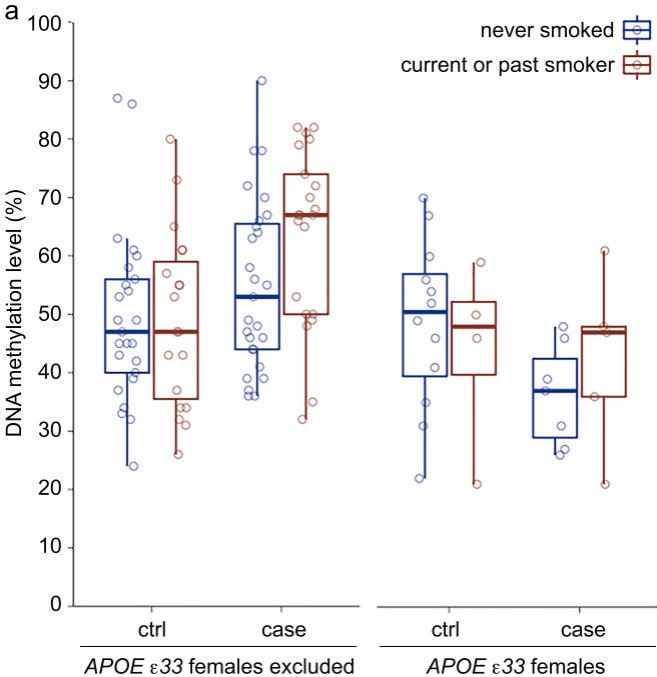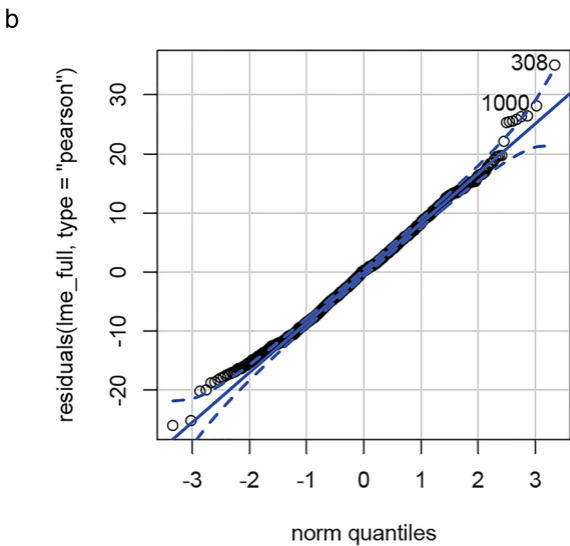

Supplement: Supplementary file 9 — DNA methylation level of the ADARB2 mark is higher in the Alzheimer’s disease cases with a history of smoking. Smoking history was included in the linear mixed effects model as a fixed effect, and interaction term, to determine whether this adjustment attenuates association of the ADARB2 DNA methylation mark with Alzheimer’s disease. The analysis was carried out by including all the 60 twin pairs with smoking data available. According to the results the disease association remained significant also when smoking was included in the model (estimate 64.51%, SE 9.23, Wald t value 6.99, not adjusted for APOE genotype). Smoking did interact with the disease status (estimate 8.10%, SE 1.43, Wald t value 5.68) and the median methylation level was increased in the cases with a smoking history in comparison to non-smoking cases and controls as well as controls with a smoking history. The association remained significant also when APOE genotype was included in the model (estimate 35.41%, SE 9.41 and Wald t value 3.76). A) DNA methylation levels for the ADARB2 mark in non-smokers and past or current smokers are shown separately for females with APOE ε33 genotype who differ from the other groups and show no disease association. B) Residual plot for the full model: me ~ dis + dis * zyg + dis * age + dis * sex + dis * smo + dis * apoe + (1 | pairid/pos). me = CpG methylation level, dis = disease status, zyg = zygosity, smo = smoking history (never, ever), apoe = apoe genotype group, pairid = twin pair information, pos = genomic position. (PDF 715 kb) [file 13148_2019_729_MOESM9_ESM.pdf]
